# Association of pro-inflammatory cytokines with trauma and post-traumatic stress disorder visiting a tertiary care hospital in Kathmandu

**DOI:** 10.1371/journal.pone.0281125

**Published:** 2023-02-02

**Authors:** Rishav Koirala, Hans Christian D. Aass, Erik Ganesh Iyer Søegaard, Hari Prasad Dhakal, Saroj Prasad Ojha, Edvard Hauff, Suraj Bahadur Thapa

**Affiliations:** 1 Division of Mental Health and Addiction, Institute of Clinical Medicine, University of Oslo, Oslo, Norway; 2 Brain and Neuroscience Center, Nepal; 3 Department of Medical Biochemistry, Oslo University Hospital, Oslo, Norway; 4 Division of Mental Health and Addiction, Oslo University Hospital, Oslo, Norway; 5 Department of Pathology and Laboratory Medicine, Nepal Cancer Hospital and Research Center, Nepal; 6 Department of Psychiatry and Mental Health, IOM, TUTH, Nepal; Chiba Daigaku, JAPAN

## Abstract

Post-traumatic stress disorder (PTSD) is a mental disorder that can occur after trauma. Although inflammatory markers such as cytokines are found altered in trauma and PTSD, there is no consensus regarding which can be considered as biomarkers. Studies from South Asia region is also rare. We studied cytokines among trauma affected patients and matched healthy controls. Fifty patients (cases) with trauma, visiting the University hospital in Kathmandu and thirty-nine healthy controls were selected, and the levels of cytokines were determined using a Luminex IS 200. We compared the levels of the cytokines in thirty-four age and gender matched pairs of case and control among three groups: healthy volunteers, cases diagnosed as PTSD, and cases without PTSD. Among the 34 pair-matched cases and controls, IL-6 was significantly higher in both PTSD positive cases [2.43 (0.00–14.54) pg/ml; p = 0.004] and PTSD negative cases [3.00 (0.92–3.86) pg/ml; p = 0.005], than in controls [0.39 (0.00–11.38) pg/ml]. IL-1β was significantly higher in PTSD positive cases [0.17 (0.00–5.27) pg/ml; p = 0.011] than in controls 0.00 (0.00–0.12) pg/ml. Other cytokines did not show significant differences. IL-6 was higher in both the trauma affected groups and IL-1β was higher in the trauma affected group with PTSD when compared to healthy controls. This supports the immune system activation hypothesis after trauma.

## Introduction

Post-Traumatic Stress Disorder (PTSD) is a chronic debilitating mental disorder that occurs in some of the people who have been exposed to traumatic events. The recent World Mental Health Survey has shown that the prevalence of PTSD varies from 0.3% to 8.8% in the general population and 0.5% to 14.5% in trauma exposed populations [1]. The prevalence rate of PTSD in the general population/community of South Asia was seen in a systematic review and meta-regression analysis to be 13.05% [2], which is seen to be higher than the prevalence of PTSD as seen in the Western countries such as USA (8.3%), Australia (9.6%) and France (5.4%) [1]. These differences may also be due to methodological differences such as study design, questionnaires used etc.

According to The International Classification of Diseases 10^th^ Revision (ICD-10), PTSD has been defined by three clusters of symptoms that develop in response to trauma, namely 1) avoidance of the stressor 2) reexperiencing/reliving the moment of trauma and, 3) either one of these: increased psychological or physiological sensitivity or amnesia of the traumatic period [3]. These symptom clusters are the basis for clinicians and researchers to identify and diagnose PTSD.

Identifying biomarkers in patients with PTSD, will not only provide a more precise diagnostic assessment but may also help to evaluate the severity of PTSD. The combination of symptom assessment and evaluation of biochemical markers will not only give better criteria for diagnostics but may also suggest more refined treatment options.

Several studies have reported increasing evidence of neurobiological findings in this disorder. Morphological changes in the hippocampus, amygdala, and prefrontal cortex among PTSD patients have been found by several imaging studies [4, 5]. Association of genes with PTSD is another biological aspect that has received increased interest in research in the last decade with robust findings [6, 7]. The role of inflammation and activation in the immune system has been another focus in the search for neurobiological consequences leading to PTSD [8–10].

Cytokines have been explored as a biomarker for mental disorders [11–13]. Cytokines are one of the most studied biomarkers and it may be valuable in diagnosing and monitoring PTSD in the future [8, 11]. Activation of the immune system in PTSD patients has repeatedly been shown [8, 14]. Furthermore, a close association or comorbidity of PTSD with physical illnesses and abnormalities such as obesity [15], cardiovascular diseases [16], diabetes [17], autoimmune diseases [18], and accelerated aging [19] also may indicate a role of immune dysregulation in PTSD.

Traumatic experiences have shown to bring changes in the immune system, even in those who don’t develop PTSD [20–22]. In PTSD patients, however, these findings seem to persist [23]. A temporarily unbalanced immune response would probably not cause any mental disorders including PTSD. However, an escalated and persistent inflammatory response may contribute to some of the symptoms observed in PTSD patients [24]. It may be difficult to identify the trigger (or source) of neurological biomarkers release, as immune activation may be triggered not only by PTSD but by other associated somatic factors such as aging, cardiovascular disease, diabetes, etc.

One hypothesis proposed by researchers is that PTSD is an immune related disorder that occurs in response to trauma [8, 25]. Several studies have observed increased levels of pro-inflammatory mediators in individuals diagnosed with PTSD [26, 27]. The most studied immune markers are pro- and anti-inflammatory cytokines. Pro-inflammatory cytokines (e.g., IL-1β, TNF-α, IL-2, IL-6, IL-8, IL-17, IFN-ϒ, IL-2RA, etc.) are expressed and secreted upon cellular stimuli, bind to receptors on target immune cells, and by this, elicit tailored inflammatory immune responses, such as recruitment and activation of other immune cells, clearing of damaged tissue and repair, induce expression of other cytokines, and cause the release of reactive oxygen species (ROS). The anti-inflammatory cytokines (e.g., IL-4, IL-10, IL-13, etc.), on the other hand, serve as important modulators to balance immune response and may dampen the effect of those that drive inflammation [28].

Elevated levels of both the pro-inflammatory cytokines such as IL1-β, IL-6 and TNF-α [27] and anti-inflammatory cytokines such as IL-1RA [23] have been observed in patients with PTSD compared to healthy individuals. Among several pro-inflammatory biomarkers, IL-1β and IL-6, play a dominant role in the cause of destructive modifications in the body, once up-regulated [29, 30]. However, these destructive changes are balanced by the anti-inflammatory cytokines such as IL-10 and IL-1RA when such immune system activation occurs [31, 32]. Cytokine levels have also been seen to be influenced by other multiple factors such as age, gender, alcohol, smoking and drug abuse, and other physical and mental illnesses [33, 34]. Although there have been several studies investigating the role of cytokines in PTSD, the results are not consistent [35–38]. Even the most commonly studied cytokines in context to PTSD such as IL-6 [27, 39–42], IL-1β [41–43] and TNF-α [41, 43, 44] has shown diverse findings. Most importantly the data regarding the cytokines are mostly from the western and developed countries [36, 37, 45], hence there is a gap with context to data from the underdeveloped countries. We came across only one research done exploring PTSD and cytokines in the South-Asian context [40] and very few in Asian region. Our study aims to fill this gap too.

In this study, our objective was to compare the plasma levels of both pro- and anti-inflammatory cytokines between trauma affected patients and, in an age- and gender-matched healthy control group, and further assess whether higher cytokine levels are associated with PTSD diagnosis.

## Materials and methods

### Study population

This is a comparative study with a cross-sectional design. One hundred and two study participants were selected from the outpatient department (OPD) of the Department of Psychiatry and Mental Health of Institute of Medicine (IOM), Tribhuvan University Teaching Hospital (TUTH), Kathmandu, Nepal. The details regarding the sample size calculation can be accessed from the previous article related to the same data [46]. Among the 102 participants included in our study, two did not meet the inclusion criteria and only 50 of them came on the next day of their interview to give their blood sample for analysis. This low presence might have happened as culturally people in this region are hesitant to give blood for research purpose and another reason may be because most of the participants were form outside Kathmandu, where the hospital is situated, that would prolong their duration of stay and hence leading to increased cost. All the patients were informed about the research and their rights to remove their consent any time they wished. All the literate patient signed a written consent paper while those who were illiterate the consent paper was read to them and asked if they could understand it all, the person accompanying them gave the written consent on their behalf. Patients between the age of 18 to 60 years and having a history of trauma were recruited in this study. Patients with serious mental illnesses such as psychosis, bipolar affective disorder were excluded from the study. Patients who were physically seriously ill with acute physical problems, whose vitals were unstable were excluded from the study. Further details about the patients and their characteristics is provided in a previous research paper exploring the same sample [47].

Thirty-nine healthy volunteers (control group, here onwards) were selected randomly among healthy blood donors at the blood bank of Oslo University Hospital. They filled out a self-report form where they confirm that they do not have a history of mental illness or major physical diseases.

To avoid biases in the value of cytokine due to age and gender, we did one to one matching between cases and controls for gender and age (±5 years). After the matching, the final number was 34 pairs of cases and controls.

### Recruitment procedure and data collection

After the interview for the evaluation of sociodemographic profiles and diagnosis of PTSD, the patients in our study were asked to come fasting to the laboratory the next morning. The blood samples were collected between 9:00 am to 10:00 am. Four mL of antecubital venous blood was collected in an EDTA tube. Within 30 minutes of drawing the blood, the plasma was separated on a swing-out centrifuge (2000xg) at 4°C and transferred into 3 polypropylene tube, which was then stored at -80°C. The samples were transported in dry ice to the Oslo University Hospital in Norway.

### Questionnaires

#### Socio-demographic questionnaire

This consisted of a question on socio-demographic characteristics such as age, gender, marital status, socioeconomic status (SES), and education for the patients in our study. Kuppuswami socioeconomic scale, a widely used scale in the South-Asian region, was used to assess SES [48]. It has been modified to fit the Nepali context [49].

#### The composite international diagnostic interview 2.1 (CIDI)

The CIDI is a comprehensive, standardized diagnostic interview designed for assessing mental disorders according to the definitions of the Diagnostic Criteria for Research of ICD-10 [50]. CIDI version 2.1 has been translated and validated in Nepali [51]. Section K of CIDI 2.1 was used to diagnose PTSD. Section K also includes a list of ten possible traumatic events along with a question to specify the primary trauma.

#### Quantification of plasma cytokine levels

A custom 7 plex (cat. no.: 17007417, Bio-Rad Laboratories, Inc.) was used to determine plasma levels of IL-1 β, IL-1RA, IL-2, IL-2RA, IL-6, MIF, and TNF- α in patients and healthy volunteer samples. Samples were thawed, vortexed and kept on ice until centrifugation at 10 000xg/10min/4°C. The supernatant was further diluted (1+3) in separate tubes using the recommended sample diluent reagent and vortexed. Fifty microliters of the sample was loaded onto assay plate, whereas all samples were analysed in duplicate. The assay was optimized for “low level detection” by adding a standard point and extending the incubation of multi-plex beads and sample for up to one hour. All washing steps were performed with an automated magnetic plate washer (Bio-plex Pro wash station, Bio-Rad Laboratories, Inc. Hercules, CA, USA). The cytokine levels were determined using a Luminex IS200 instrument (Bio-Rad Laboratories, Inc. Hercules, CA, USA). A spiked plasma sample was used as a control to determine the percentage coefficient of variation (%CV). Intra %CV ranged from 0.5–10 and plate to plate variation (inter %CV) ranged from 5–19.

#### Statistical analysis

SPSS version 27.0 was used for the analysis. Data was initially checked for normal distribution with the Kolmogorov-Smirnov test. Since all of the variables except MIF did not follow the normal distribution, nonparametric test, the Independent-samples median test was used to compare median cytokine levels between the three groups; controls, cases with PTSD, and cases without PTSD. Furthermore, post hoc Bonferroni correction was done on the pairwise data for cytokines that showed significance between the three groups to find pairwise relation of cytokines among the three groups.

#### Ethical consideration

The study was approved by the Institutional Review Board of IOM, TUTH reference no 278 (6-11-E) 073/074; Nepal Health Research Council (NHRC), reference no 801 and Regional Ethical Committee of South-Eastern Norway (REK Sør-Øst), reference no. REK 2015/2081.

## Results

Since there was no cytokine data for half (n = 50) of the total patients (n = 100), we did an equivalence test between the two groups using the chi-square test to see if there was any difference in the two groups in terms of the studied variables, age, gender and PTSD diagnosis. There was no statistically significant difference between the groups.

The sociodemographic profile of the total and matched cases from our study population is given below in Table 1.

**Table 1:** Sociodemographic profile of total and matched cases.

Variable		Total cases (%) N^1^ = 50	Matched cases(%) N = 34
Age	18–30 years	21 (42%)	9 (26.5%)
	31–45 years	21 (42%)	18 (52.9%)
	46–60 years	8 (16%)	7 (20.6%)
Gender	Female	28 (56%)	19 (55.9%)
	Male	22 (44%)	15 (44.1%)
Relationship status	Single	10 (20%)	3 (8.8%)
	Married	39 (78%)	30 (88.2%)
	Divorced	1 (2%)	1 (2.9%)
Socioeconomic Status	Upper class	7 (14%)	5 (14.7%)
	Upper middle class	28 (56%)	19 (55.9%)
	Lower middle class	12 (24%)	8 (23.5%)
	Lower class	3 (6%)	2 (5.9%)
Education	Illiterate	7 (14%)	5 (14.7%)
	Up to high-school	29 (58%)	21 (61.8%)
	Above high-school	14 (28%)	8 (23.5%)

^1^ Total number

Further details on the sociodemographic background of the study populations have been described earlier [47].

The Independent samples median test was done on the total cases (n = 50) and controls (n = 39) on forming the three groups: healthy volunteers (control group) (n = 39), cases with PTSD (trauma + PTSD group) (n = 42) and cases without PTSD (trauma—PTSD group) (n = 8). Values of IL-2 were below detection range and not computable. The values of IL-6 (p = 0.006), MIF (p = 0.020) and TNF-α (p = 0.003) were significantly higher in cases than controls.

These three cytokines, which showed significant differences in the full sample, were further analyzed using the post hoc Bonferroni correction, a pairwise test to find the individual relationship within the three groups. In this analysis, IL-6 levels (median value followed by interquartile range) were significantly higher in trauma cases without PTSD [2.58 (1.22–3.54) pg/ml; p = 0.003] than controls 0.39 (0.00–1.45) pg/ml. MIF was significantly higher in both PTSD positive cases [2476.8 (1890.3–2913.5) pg/ml; p = 0.016] and PTSD negative cases [2537.36 (2094.69–3222.42) pg/ml; p = 0.050] than in the controls 1856.3 (1540.6–2324.3) pg/ml. TNF-α was significantly higher in both PTSD positive cases [45.14 (28.98–55.95) pg/ml; p = 0.030] and PTSD negative cases [57.28 (52.70–63.07) pg/ml; p = 0.050] than the controls 28.06 (21.62–39.91) pg/ml.

Table 2 lists total cases (n = 50) and controls (n = 39) and pairwise matched cases (n = 34) and controls (n = 34) on age, gender, and PTSD diagnostic status. The median age for matched males was 36 years for both cases and controls and 34 years and 35 years for females of cases and controls, respectively. Among the 34 control-paired cases, 80% had met the criteria for PTSD and 20% did not.

**Table 2:** Comparison of general characteristics of the full sample and matched pair samples.

Variables		Total (89)		Matched (34 pairs)	
		**Cases (50)**	**Controls (39)**	**Cases (34)**	**Controls (34)**
Gender	Male	22	15	15	15
	Female	28	24	19	19
Median Age in years (IQR*)	Male	35.00 (26.25–45.25)	36.00 (28.00–45.00)	36 (27.0–42.0)	36 (45.0–28.0)
	Female	30.50 (24.25–37.00)	38.00 (33.25–46.50)	34 (28.0–41.0)	35 (28.0–44.0)
PTSD	Male	19	0	13	0
	Female	23	0	14	0

* = Interquartile Range

To further limit the chance of bias in cytokine values sourced by differences in age and gender, we performed the Independent samples median test on 34 healthy volunteers matched with 34 cases (Table 3).

**Table 3:** Differences in cytokines of pairwise matched cases, with and without PTSD, and controls, using the Independent samples median test and post-hoc pairwise analysis.

Variables	Controls (n* = 34)	Trauma + PTSD (n = 27)	Trauma—PTSD (n = 7)	2-sided exact significance (p-value)	Effect size (Cramer’s V)
	Median (IQR^#^) pg/ml^!^	Median (IQR) pg/ml	Median (IQR) pg/ml		
Age (years)	36 (25–56)	35 (24–57)	36 (27–52)	0.968	0.659
IL-1β	0.000 (0.00–0.12)	0.17 (0.00–5.27)	0.22 (0.00–0.52)	**0.002**	0.592
IL-6	0.39 (0.00–11.38)	2.43(0.00–14.54)	3.00 (0.92–3.86)	**0.003**	0.796
MIF	1883.57 (3765.64–672.89)	2528.00 (810.97–4975.59)	2639.02 (1166.03–3762.03)	**0.049**	1.000
TNF-α	30.15 (12.32–65.15)	46.97 (4.58–146.9)	56.75 (19.77–82.31)	**0.024**	0.936
IL1-RA	145.02 (0.00–1375.19)	270.38 (0.00–1825.81)	250.81 (121.49–348.24)	0.623	0.899
IL2-RA	115.31 (66.52–179.54)	136.96 (73.54–424.21)	124.31 (104.65–198.06)	0.262	0.981
**Post-hoc pairwise analysis**					
Cytokine	Sample1- Sample 2		Test Statistics	Significance	Adjusted Significance
IL-6	Control vs Trauma + PTSD		10.12	.001	**.004**
	Control vs Trauma—PTSD		9.77	.002	**.005**
	Trauma + PTSD vs Trauma—PTSD		.18	.671	1.00
IL1β	Control vs Trauma + PTSD		8.41	.004	**.011**
	Control vs Trauma—PTSD		5.26	.022	.065
	Trauma + PTSD vs Trauma—PTSD		.18	.671	1.00

* = total number

# = Interquartile Range

! = picogram/milliliter

The analysis was done on the same three groups: healthy volunteers (control group) (n = 34), cases with PTSD (trauma + PTSD group) (n = 27), and cases without PTSD (trauma–PTSD group) (n = 7). Values of IL-2 were below detection range and not computable.

The cytokines which showed significance, IL1-β (p = 0.002), IL6 (p = 0.003), MIF (p = 0.049), and TNF α (p = 0.024) were further analyzed using the post hoc pairwise test to find the individual relationship within the three groups (Table 3).

**Fig 1:**
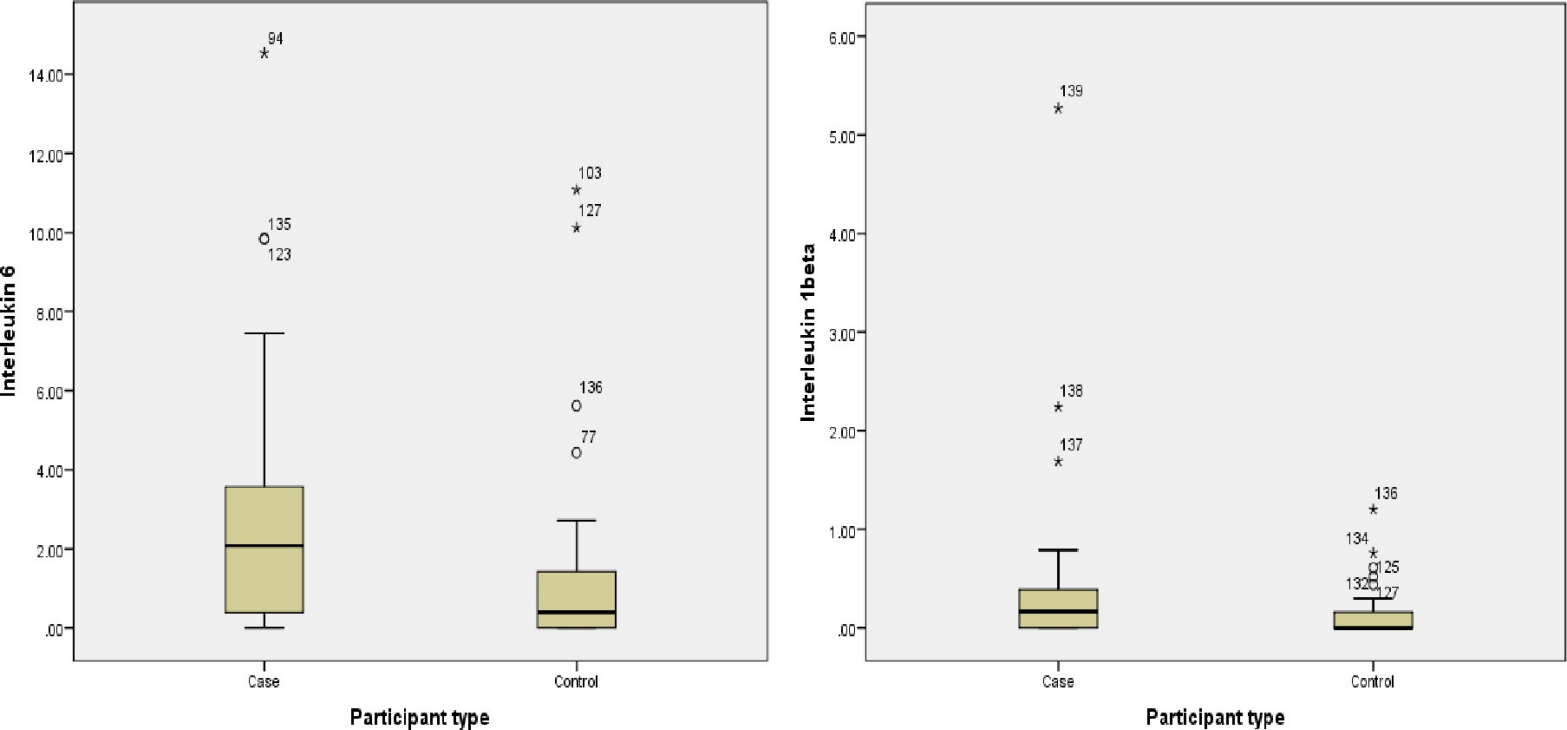
Comparison of median values of IL-6 and IL1-β between cases and healthy controls.

In the pairwise analysis (Fig 1), IL-6 was significantly higher in both PTSD positive cases [2.43 (0.00–14.54) pg/ml; p = 0.004] and PTSD negative cases [3.00 (0.92–3.86) pg/ml; p = 0.005], than in controls 0.39 (0.00–11.38) pg/ml. IL1-β was significantly higher in PTSD positive cases [0.17(0.00–5.27) pg/ml; p = 0.011] than in controls 0.00(0.00–0.12) pg/ml. MIF and TNF α did not show any significant differences in post hoc pairwise analysis.

## Discussion

In our study, we found that IL-6 plasma levels were significantly higher in trauma affected individuals, both with or without PTSD, compared to the healthy controls. This observation was supported by the Independent- samples median test on directly matched 34 samples and in the post hoc pairwise analysis. The generally matched sample also showed significantly higher levels of MIF and TNF-α for cases both with and without PTSD compared to controls. This was, however, not significant in the directly matched sample in the post hoc pairwise analysis. Further, we observed that IL1-β was significantly higher in the individuals with PTSD than the healthy controls.

In our study, IL-6 was significantly higher in the trauma exposed cases than in the healthy control group. There was, however no statistically significant difference in IL-6 levels between the PTSD and non-PTSD groups. In previous studies, it has been shown that IL-6 increases with acute trauma as well as childhood trauma [41, 42, 44, 52]. In contrast to our findings, some studies have found an increase in IL-6 levels in PTSD patients in comparison to trauma-affected non-PTSD patients [27, 43]. Similarly, some studies have not shown any changes in levels of IL-6 in relation to PTSD [38–40] or with trauma exposure [39, 40]. Interestingly, in a study by Bruenig *et al*., (2018), showed that IL-6 levels decreased in PTSD patients when compared to non-PTSD patients [35]. This may be because IL-6 at times also acts as anti-inflammatory myokine [53]. IL-6 has also been seen to be affected in major depressive disorders [54], that may have caused bias in the result. The exact role of IL-6 still needs further exploration in context to trauma and PTSD.

IL-1β is one of the major cytokines that has been explored in response to trauma and PTSD. There are several studies showing a significant rise in the level of IL-1β in both trauma-affected population [41, 42] as well as in PTSD cases [43]. A recent meta-analysis done by Passos et al. including 20 studies from more than 8000 abstracts has shown increased levels of IL-1β in trauma affected PTSD patients in comparison to the normal population [27]. In our study, IL-1β was found to be significantly higher in PTSD positive cases than the healthy controls. However, no differences were observed between PTSD positive and negative cases. Our small sample size, particularly of cases without PTSD, might be one possible reason for this. It should be kept in mind that the values of the IL-1β became significant only after the age- and gender-matched case and control were compared. This also points that it is also important to consider other factors that may lead to biases such as body mass index and smoking that were not accounted for in this study.

TNF-α is another cytokine that has shown elevated in both individuals exposed to trauma [41, 44] as well as individuals diagnosed with PTSD [27, 43]. In our Independent samples median test, TNF-α showed a significant higher level, however, with post hoc Bonferroni correction, TNF-α did not show any significant differences among the three groups. This may have been due to the small sample size of the PTSD positive cases. Supporting our findings, a previous study done in Nepal also didn’t find any relationship between TNF-α and trauma or PTSD [40]. Surprisingly, another study done on a large Vietnam veteran cohort has shown TNF-α to decrease in PTSD population in comparison to non- PTSD population [35].

Other pro-inflammatory cytokines such as IL-2 and IL-2RA, did not show any significant difference with either experience of trauma or presence of PTSD diagnosis. These findings are in line with several studies that have been carried out previously [40]. Our study did not demonstrate any relation of TNF-α with either trauma experience or PTSD diagnosis. This finding is in line with the results of a study done in Nepal previously [40]. But research done in other parts of the world has shown a positive relation of TNF-α levels with trauma experience [41] as well as PTSD diagnosis [27, 55]. In the current study, IL-1R, an anti-inflammatory cytokine did not show any relation with either trauma experience or PTSD.

The variations in the level of cytokines seen in context to PTSD and trauma indicate that several factors causing biases and influencing the results including small sample size still need to be explored. Several factors like age, gender, body mass index, time of trauma, alcohol, smoking and substance abuse, chronic illnesses, medication use, etc. need to be considered. Another point may be that PTSD diagnosis as such might not be related to increased cytokines but rather specific symptoms or symptom cluster of PTSD might have a relation with particular cytokines. Hence, further exploration of symptom clusters of PTSD with a particular cytokine might help us understand the relationship between psychological distress and inflammation with more consistent findings. Thus, further research in this direction is needed.

### Strengths and limitations

This is one of the very few studies on cytokines and PTSD being done in the South Asian region. The interview, as well as the sample collection, was handled by a psychiatrist. We have attempted to address biases in the cytokine levels due to age, gender, and comorbidity of major physical illnesses. Still, there are several shortcomings in this research. Other factors such as smoking, alcohol, and some stable but chronic conditions such as hypertension, diabetes were not considered. Few of the cases were taking analgesics and antidepressants, which may have also influenced the cytokine levels. The sample size was small and comorbidities such as depression and generalized anxiety disorder were prevalent among the patient population. As mental illnesses such as depression [56–58] and anxiety [59] have also shown relation with cytokines in several studies that may also have created biases in the result. Since this is a cross-sectional study, we cannot confirm a causal direction or timing of different cytokine levels, and trauma and PTSD. We also have used a control group from Norway; hence they might have created some biases due to difference in ethnicities. Some of the specific steps regarding timing of blood collection, thorough examination of physical and mental illness was not known in case of the healthy control in the way done for the cases. This too may have added to biases in the results.

## Conclusions

IL-6 levels were higher in the trauma affected population and IL-1β was higher in the trauma affected PTSD patient population in comparison to healthy volunteers. The results of our study indicate that there is an association of pro-inflammatory cytokines in response to trauma. It supports the hypothesis of immune system activation in trauma.

Further studies are needed to elucidate the importance of IL-1β and IL-6 in trauma exposed individuals and its role in PTSD. In designing new studies, a larger sample size with mapping possible bias, such as alcohol consumption, smoking habits, evaluation of history, and current state of somatic and mental illness, will be important to consider.

## Supporting information

S1 Data (SAV)
